# Molecular Dynamics-Based Calibrated Micromechanics Model for Elastic Properties of Fullerene-PMMA Nanocomposites Incorporating Interface Stress

**DOI:** 10.3390/molecules31060944

**Published:** 2026-03-12

**Authors:** Saeid Sahmani, Eligiusz Postek, Tomasz Sadowski

**Affiliations:** 1 Institute of Fundamental Technological Research, Polish Academy of Sciences, Pawińskiego 5B, 02-106 Warsaw, Poland or s.sahmani@ug.edu.ge (S.S.); epostek@ippt.pan.pl (E.P.); 2 Department of Civil Engineering, School of Science and Technology, The University of Georgia, Tbilisi 0171, Georgia; 3 School of Civil, Environmental, and Architectural Engineering, Korea University, Seoul 02841, Republic of Korea; 4 Department of Solid Mechanics, Lublin University of Technology, 20-618 Lublin, Poland

**Keywords:** molecular dynamics simulation, nanocomposites, interface effect, micromechanics, mechanical properties

## Abstract

Fullerene-based polymer nanocomposites are promising candidates for micro- and nano-electromechanical systems (MEMSs/NEMSs) due to their tunable mechanical performance and high surface-to-volume ratios. At the nanoscale, interfacial stresses strongly influence the effective elastic response, yet quantitative interface parameters are rarely available for continuum modeling. In the current investigation, a molecular dynamics (MD)-based calibrated micromechanics framework is developed to predict the bulk modulus of fullerene-poly(methyl methacrylate) (PMMA) nanocomposites that incorporate interface stress effects. Atomistic representative volume elements (RVEs) containing individual fullerene nanoparticles embedded in a polymer matrix are generated using controlled molecular packing and systematically equilibrated. The bulk moduli of both isolated fullerenes and fullerene-PMMA RVEs are extracted from energy-volume relationships using a Birch-Murnaghan equation of state. These MD results are used to calibrate a size-dependent micromechanics model and to extract the surface Lamé modulus of the polymer-fullerene interface directly. The extracted surface Lamé modulus remains nearly constant (approximately 19 N/m) across all investigated fullerene sizes. In contrast, the interfacial contribution to the effective bulk modulus increases significantly for smaller nanoparticles due to their higher surface to volume ratios. The calibrated model accurately reproduces MD predictions and provides a physically grounded multiscale link between atomistic interfacial behavior and continuum elastic properties. The proposed framework offers a predictive tool for the rational design of surface-dominated nanocomposites in MEMS/NEMS applications.

## 1. Introduction

Advanced materials, such as nanocomposites, are excellent candidates for producing more efficient micro- and nano-electromechanical systems (MEMSs and NEMSs) and are predicted to play an essential role in the growth of a value-added production economy. Outstanding mechanical, electrical, and thermal properties make fullerene one of the most promising materials to be used as a filler in nanocomposite production [1]. A combination of the distinctive features of fullerenes with the physical properties of polymers may yield advanced polymeric materials with novel physico-mechanical characteristics [2].

Fullerene nanocomposites represent a promising new class of structural materials for the mechanical components of NEMSs and MEMSs [3,4]. The utility of fullerene-reinforced composites for designing materials with low stiffness and high natural frequencies of vibration has achieved utmost importance in technology. Polymer-based nanocomposites reinforced with dispersed fullerene are excellent candidates for micro-actuators and micro-resonators, with properties rivaling those of monolithic metallic and ceramic structures used in current-generation NEMSs and MEMSs. Fullerene-based nanocomposites have attracted significant research interest in recent years for various potential applications due to their unique mechanical, electrical, optical, and chemical properties [5,6,7,8,9,10].

On the other hand, the properties of interfaces, as well as surface stress, may have significant effects on both elastic fields and the effective moduli of nanocomposite materials because reinforcements at the nanoscale have a high interface area to volume ratio, which makes the role of interface contribution to the local stress field substantial; see [11]. Accordingly, it is essential to consider the surface stress and interface effects in analyzing the mechanical characteristics of materials and structures at the nanoscale. For example, Nazarenko et al. [12] incorporated Gurtin-Murdoch-based surface bending stiffness into the conditional moments technique to extract the effective mechanical properties of random particulate composites in the presence of surface stress effect. They proposed closed-form expressions for the effective moduli of a nanocomposite containing randomly distributed spherical nanoparticles, accounting for inhomogeneity and surface effects. Sahmani et al. [13,14,15,16] utilized Gurtin-Murdoch elasticity [17,18] to demonstrate the surface stress type of size effect on the nonlinear free oscillation response of truncated conical nanoshells with in-plane heterogeneity. In another study, Lucchetta et al. [19] performed a molecular dynamics simulation to predict the overall strength of nanocomposites reinforced with nanoparticles of different sizes in the presence of surface stresses at the matrix/inclusion interface. Therefore, to capture the mechanical properties of nanocomposites as accurately as possible, it is necessary to incorporate surface stress into the associated micromechanical model.

Also, within the framework of conceiving structures, employing robust analysis tools is a common way to obtain a well-established design with mechanically compliant characteristics. Accordingly, discovering opportunities outside routine design concepts is a risky gamble that engineering companies rarely meet. In other words, structures are generally designed to exhibit high stiffness with small deformations, leading to linear behavior. In this case, there are several decades of experience to avoid undesired shape changes within nonlinear domains, which often lead to uncontrolled failure. However, if one could learn to control such a response, it would be possible to acquire the benefits of nonlinear characteristics in the design of structures. Primarily, this is of great importance for microstructures, where responses are size-dependent; the design space is usually limited; and an optimized, predictable structure would remove important technical challenges. For instance, Ghayesh et al. [20] analyzed the size-dependent nonlinear electro-elastic–mechanical behavior of MEMSs with initially curved, deformable electrodes at high AC frequencies. In another work, Lyu et al. [21] combined the benefits of mode localization and nonlinear dynamics to propose an ultrasensitive MEMS mass sensor with two electrostatic-coupled microbeams. It was shown that sensitivity can be significantly enhanced when the device is driven beyond its critical Duffing amplitude (nonlinear deformation) compared to the linear regime. Barakat et al. [22] predicted the mechanical properties of heterogeneously structured glassy polymer nanocomposites using molecular dynamics (MD) simulations, in which interphase regions were modeled via a gradient in the properties. Reda et al. [23] employed MD simulations to investigate the role of polymer chain conformations in the reinforcement characteristics of glassy polymer nanocomposites.

Conversely, size-dependent continuum models typically introduce one or more intrinsic length-scale parameters that must be calibrated to capture physical size effects. However, such parameters are not available from standard experiments alone and are increasingly calibrated using MD simulations that provide atomistically informed responses for small-scale materials and structures [24]. These MD outputs enable fitting continuum model responses (such as stiffness versus size), thereby determining length-scale coefficients or nonlocal kernel parameters [25,26,27]. Several methodological families have emerged: direct curve-fitting of continuum responses to MD data; dispersion-based matching whereby MD-derived dispersion is fit to continuum dispersion relations; inverse- or Bayesian-optimization methods for parameter identification (including uncertainty quantification); hierarchical multiscale or coarse-graining approaches; and, more recently, machine learning-augmented pipelines. These techniques have been successfully applied to nanobeams, nanotubes, and nanoplates, demonstrating that calibrated continuum models can efficiently reproduce MD behavior [24]. In recent years, several explorations have been carried out in this regard, focusing on improving the robustness and generality of the calibration process and establishing physically consistent multiscale frameworks that seamlessly integrate atomistic insights into continuum formulations for the predictive modeling of nanoscale mechanical behavior, such as [28,29,30,31,32,33,34,35,36].

The existing literature reveals that while numerous continuum and micromechanics frameworks have been developed to capture size-dependent effects in nanostructured materials, most rely on phenomenological parameters whose physical meaning and accuracy remain uncertain without atomistic calibration. Furthermore, few studies have explicitly addressed how interfacial stresses influence the effective elastic response of polymer nanocomposites reinforced with molecular-scale inclusions such as fullerenes. Given the prominent role of surface effects at the nanoscale, especially in systems with high surface to volume ratios, there remains a critical need for a rigorously calibrated model that links atomistic interfacial behavior to macroscopic elastic properties. The present work addresses this gap by developing an MD-based calibrated micromechanics model that explicitly incorporates interface stress to predict bulk moduli for fullerene-poly(methyl methacrylate) (PMMA) nanocomposites. Fullerenes are employed in this study as idealized nanoscale reinforcements rather than as direct industrial filler candidates. Their precisely defined atomic structure, uniform curvature, and absence of morphological defects provide a well-controlled platform for investigating curvature-dependent interfacial mechanics and surface stress effects at the molecular scale. This eliminates ambiguity associated with irregular particle geometry, surface roughness, or agglomeration, which are commonly encountered in practical nanofillers. The primary objective of this work is, therefore, methodological development and multiscale model calibration rather than the optimization of a specific composite formulation.

This integration of MD-derived interfacial parameters with an analytical continuum framework not only ensures physical consistency across scales but also enhances the predictive reliability of continuum models for the design and optimization of next-generation MEMS/NEMS components. In other words, the novelty of the current study lies not in demonstrating generic filler reinforcement, which has been extensively reported for polymer nanocomposites, but in quantitatively calibrating a surface stress-enhanced micromechanics framework directly from MD simulations. By extracting the physically meaningful interfacial elastic parameters and demonstrating their size invariance across multiple nanoparticle diameters, the proposed approach establishes a rigorous multiscale link between atomistic interfacial behavior and continuum elastic predictions. This capability enables the predictive modeling of surface-dominated nanocomposites beyond a phenomenological fitting.

## 2. Results

### 2.1. Extracted Bulk Modulus of Fullerene Nanoparticles

The bulk modulus values for the considered fullerenes with various diameters are determined using MD simulations and an equation of state (EOS) fitting approach. The methodology is implemented in the Large-Scale Atomic/Molecular Massively Parallel Simulator (LAMMPS) [37,38], with the carbon-carbon interactions modeled using the Adaptive Intermolecular Reactive Empirical Bond Order (AIREBO) potential [39]. This potential function is well suited for sp^2^-bonded carbon systems, as it incorporates both short-range covalent interactions and long-range van der Waals contributions.

The initial step involved preparing a relaxed configuration of each fullerene cage. The atomic coordinates are minimized by a conjugate gradient until the forces on all atoms and the total potential energy converge to within a predefined tolerance. Once the relaxed structure is obtained for each of the $C_{24}$, $C_{36}$, $C_{44}$, $C_{60}$, $C_{72}$, and $C_{84}$ fullerene nanoparticles, isotropic volumetric deformations are applied by scaling the simulation box along the three Cartesian directions. In LAMMPS, this can be accomplished via the *remap* command, which ensures that both the box dimensions and atomic coordinates are rescaled consistently.

**Figure 1:**
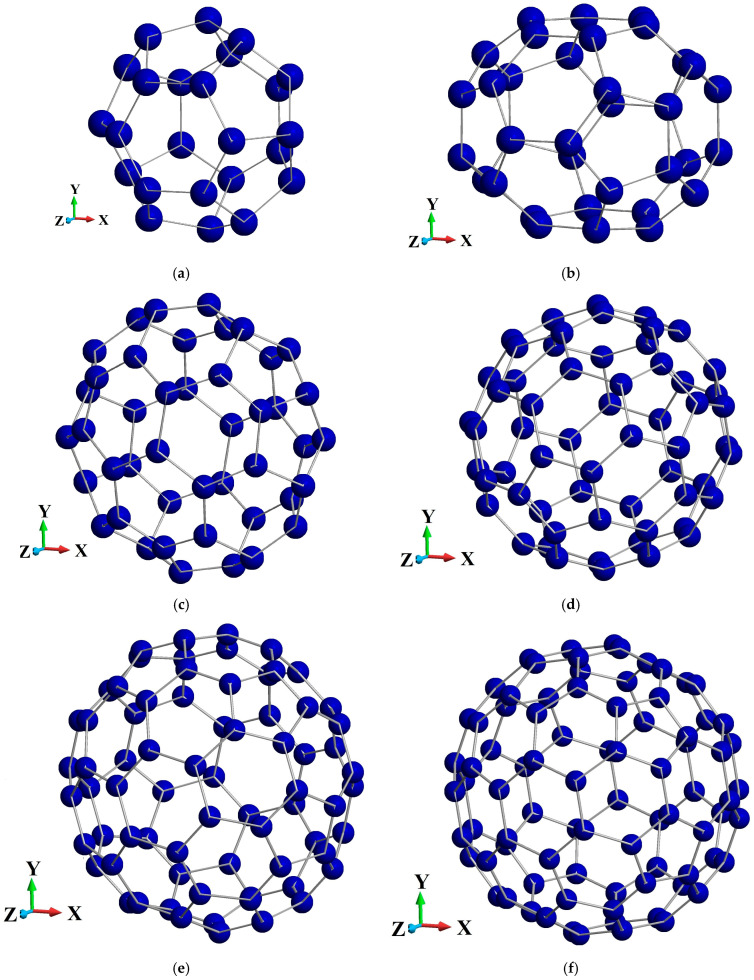
Atomistic models of considered fullerene nanoparticles: (**a**) C_24_; (**b**) C_36_; (**c**) C_44_; (**d**) C_60_; (**e**) C_72_; (**f**) C_84_.

A series of positive (tensile) and negative (compressive) volumetric strain states is imposed around the equilibrium volume, typically ranging from about −3% to +3%. After each strain increment, a complete structural minimization is performed repeatedly to remove residual stresses and ensure the system has reached a mechanically stable state. In Figure 1, the atomistic models of fullerene nanoparticles used to estimate their bulk modulus are depicted.

For every strained configuration, the system’s total potential energy, E, and corresponding effective molecule volume, V, are extracted. This yields a discrete set of (E,V) data points that serve as the basis for determining the bulk modulus. In this regard, the energy–volume relation is employed in conjunction with the EOS fitting approach.

The bulk modulus is formally defined as the second derivative of the energy with respect to volume at equilibrium, as follows:

(1) $$B=V{\left.\frac{\partial^{2}E}{\partial V^{2}}\right|}_{V=V_{0}}\;\;\;,$$

in which $V_{0}$ refers to the equilibrium volume, and E stands for the internal energy of the system. Rather than computing finite differences directly, a more robust method is to fit the (E,V) data to an analytical equation of state. In this work, the third-order Birch-Murnaghan EOS is employed, which is widely used for molecular and crystalline solids under isotropic strain. The functional form of the EOS is given as

(2) $$E\left(V\right)=E_{0}+\frac{9V_{0}B_{0}}{16}\left[{\left({\left(\frac{V_{0}}{V}\right)}^{\frac{2}{3}}-1\right)}^{3}B_{0}^{\prime }+{\left({\left(\frac{V_{0}}{V}\right)}^{\frac{2}{3}}-1\right)}^{2}\left(6-4{\left(\frac{V_{0}}{V}\right)}^{\frac{2}{3}}\right)\right]\;,$$

where $E_{0}$ is the minimum energy, $B_{0}$ signifies the bulk modulus, and $B_{0}^{\prime }$ denotes the first pressure derivative of the bulk modulus.

The fitting is performed using nonlinear least-squares optimization implemented in a MATLAB R2023a coding script. The procedure simultaneously optimized the four fitting parameters $(E_{0}$,$V_{0}$,$B_{0}$,$B_{0}^{\prime })$ to minimize the difference between the simulation data and the EOS expression. The final reported values, $B_{0}$, corresponding to each considered fullerene are captured in gigapascals (GPa), which represent the resistance of the fullerenes to isotropic compression.

In summary, the workflow consisted of:

- Generating relaxed and strained configurations of fullerenes via MD and energy minimization;
- Extracting the energy and volume dataset across a range of isotropic strains;
- Fitting the Birch-Murnaghan EOS to the data;
- Computing the equilibrium bulk modulus.

**Figure 2:**
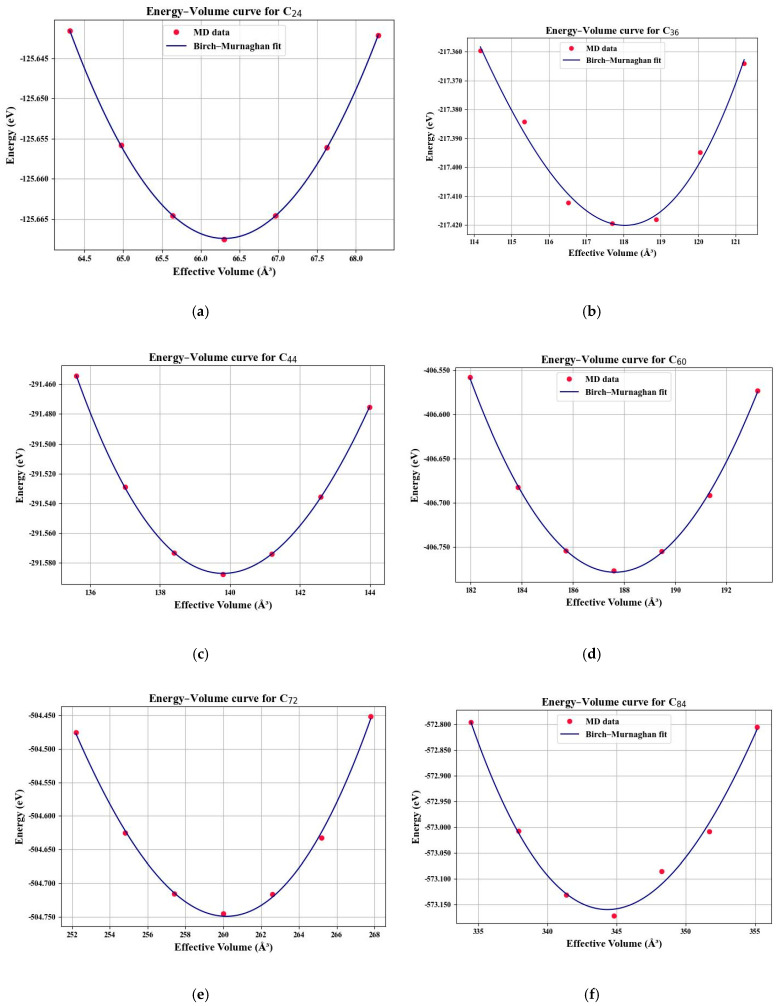
Extracted Birch–Murnaghan EOS-fitted energy–volume diagrams attributed to each fullerene nanoparticle: (**a**) C_24_; (**b**) C_36_; (**c**) C_44_; (**d**) C_60_; (**e**) C_72_; (**f**) C_84_.

This procedure provides a systematic, physically rigorous method for characterizing the intrinsic mechanical stiffness of the fullerene molecule, directly from atomistic energetics without empirical approximations. Accordingly, in Figure 2, the extracted Birch-Murnaghan EOS-fitted energy-volume diagrams attributed to each fullerene nanoparticle are demonstrated. Also, the relevant captured bulk modulus values are given in Table 1.

### 2.2. Development of the Calibrated Micromechanics Model

The quadratic curvature of the energy–volume curve at the equilibrium point directly corresponds to the hydrostatic stiffness of the RVE. Because the interface energy contribution is embedded in the total potential energy, the extracted bulk modulus represents the effective modulus of the fullerene-PMMA nanocomposites, accounting for nanoparticle–polymer interactions. Accordingly, using the Birch-Murnaghan EOS, fitted energy–volume diagrams for each fullerene nanoparticle were generated, and the relevant bulk modulus values for fullerene/PMMA nanocomposites, along with their fullerene nanoparticle volume fraction, are given in Table 2. Although the bulk modulus is conventionally reported in gigapascals, kPa units are used in Table 2 to enhance the resolution of relative differences among nanocomposite configurations.

Table 3 summarizes the calibrated values of the dimensionless size-dependent parameter $K_{s}^{*}$ and the corresponding surface Lamé modulus, $K_{s}$, for various fullerene nanoparticles embedded in PMMA. Notably, while the surface Lamé modulus remains nearly constant across all nanoparticle types (~18.96–18.98 N/m), the dimensionless parameter, $K_{s}^{*}$, exhibits a clear decreasing trend with increasing nanoparticle size. This behavior is consistent with theoretical expectations: $K_{s}^{*}$ is inversely proportional to the particle diameter, as expressed in Equation (21), and therefore, smaller nanoparticles with higher surface to volume ratios display a more substantial interfacial influence on the nanocomposite’s effective bulk modulus.

The near-constancy of $K_{s}$ across different fullerene types suggests that the intrinsic interfacial stiffness between fullerene nanoparticles and PMMA is mainly independent of particle size. This finding implies that the variation in the effective bulk modulus of the nanocomposite is primarily driven by geometric factors, namely, the relative contribution of the interface to the composite’s total volume, rather than by changes in fundamental interfacial properties. Consequently, nanocomposites reinforced with smaller fullerenes, such as $C_{24}$ and $C_{36}$, exhibit a more pronounced enhancement in bulk modulus due to the amplified effect of interfacial stress relative to larger nanoparticles.

The observed trend also underscores the importance of incorporating size-dependent interfacial effects in micromechanical modeling. Conventional models that neglect surface stress contributions may significantly underestimate the stiffness of nanocomposites containing small nanoparticles, leading to inaccurate predictions of their mechanical behavior. By calibrating $K_{s}^{*}$ using MD simulations, the proposed micromechanical framework provides a robust tool for bridging atomistic-scale interfacial phenomena with continuum-scale mechanical properties.

Moreover, the results reveal a trade-off between nanoparticle size and interfacial efficiency. While larger fullerenes occupy a higher volume fraction within the polymer matrix, the relative influence of the interface diminishes due to the lower surface-to-volume ratio, as reflected in the decreasing $K_{s}^{*}$ values. This size-dependent effect is critical for the rational design of nanocomposites, as it indicates that optimal mechanical performance can be achieved by carefully balancing nanoparticle size, volume fraction, and interfacial properties.

**Figure 3:**
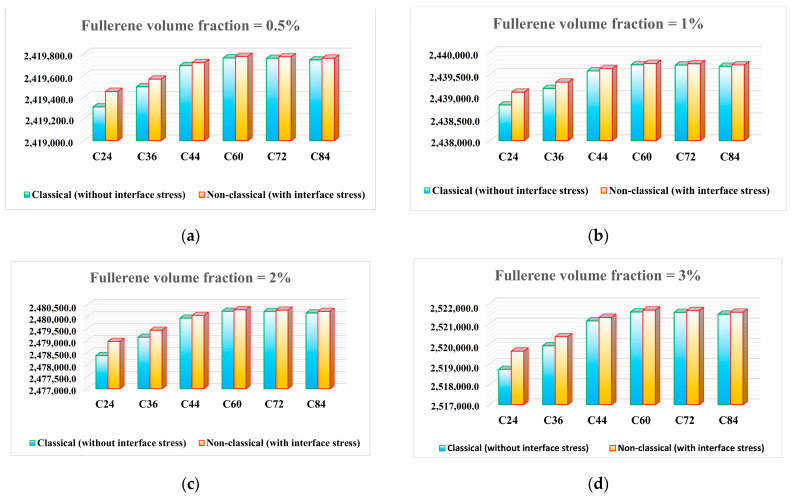
Bulk modulus (kPa) of fullerene-PMMA nanocomposites reinforced with various types of fullerenes at different volume fractions: (**a**) 0.5% volume; (**b**) 1.0% volume; (**c**) 2.0% volume; (**d**) 3.0% volume (for visualization purposes, bulk modulus values are expressed in kPa to highlight small relative differences between configurations).

Using the calibrated size-dependent model, the effective bulk modulus of fullerene–PMMA nanocomposites can be determined for any volume fraction. Accordingly, Figure 3 presents the bulk modulus values of fullerene-PMMA nanocomposites reinforced with various types of fullerenes at different volume fractions, both with and without interfacial effects considered. The results reveal that, across all volume fractions, the interfacial effect is more pronounced in nanocomposites reinforced with smaller fullerenes, reflecting the reduced contribution of surface stresses in larger nanoparticles, again due to their lower surface to volume ratios.

Overall, the present findings demonstrate that interfacial stress is a dominant factor in the nanoscale mechanical reinforcement of polymer nanocomposites. The combination of MD simulations with a size-dependent micromechanical model enables accurate predictions of bulk modulus across a range of fullerene sizes, providing valuable insights for the design of high-performance MEMS/NEMS materials.

### 2.3. Stress Observations in Fullerene Particles

In this section, the results of stress analysis of the bonds are presented. The system is relaxed as in the preceding analyses, and then, two extreme cases are considered: 2% and −2% changes in the system’s volume. The loading is applied suddenly.

**Figure 4:**
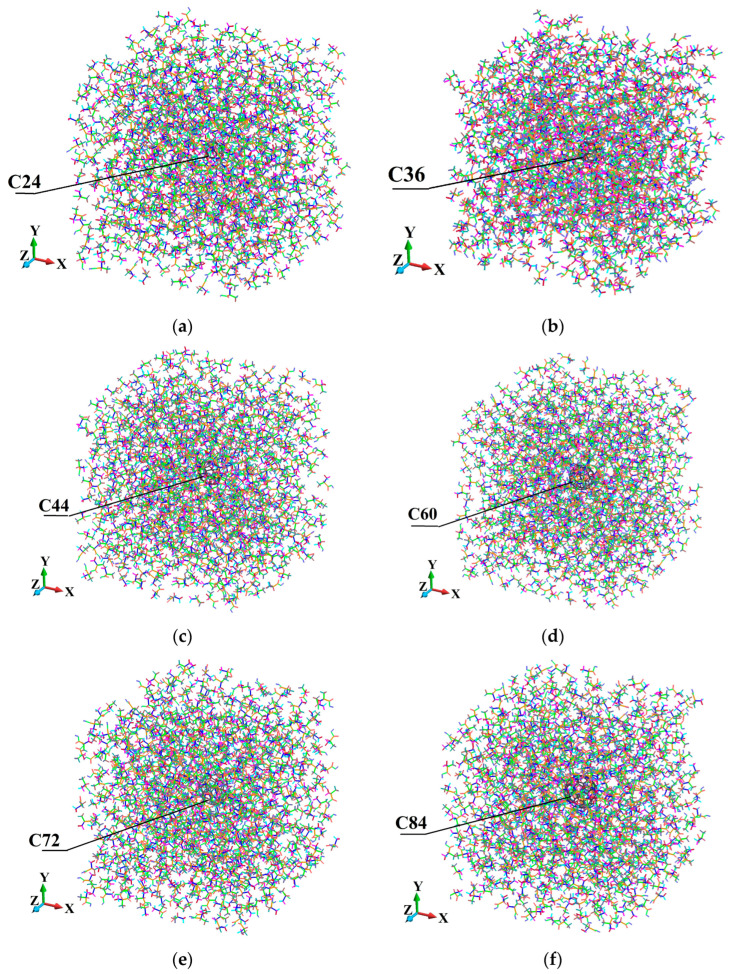
Constructed atomistic RVEs of fullerene-PMMA nanocomposites for the different fullerene types, representing consistent nanoparticle placement, polymer packing, and absence of initial atomic overlap prior to equilibration: (**a**) C_24_; (**b**) C_36_; (**c**) C_44_; (**d**) C_60_; (**e**) C_72_; (**f**) C_84_.

The axonometric view of the fullerene-PMMA systems is shown in Figure 4. There, the systems in the configuration after the relaxation procedure are presented.

**Figure 5:**
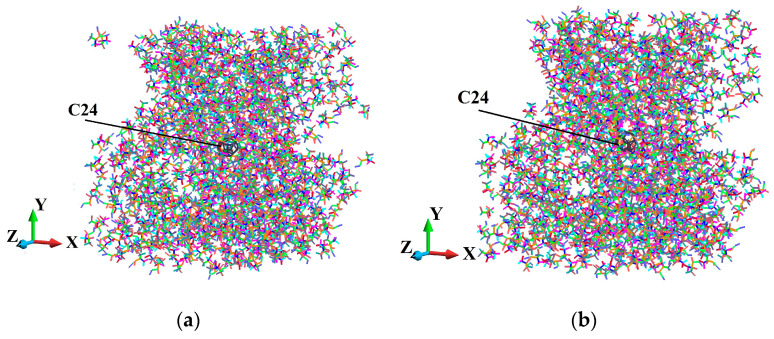
Fully relaxed RVE of C_24_/PMMA representing structural stability, consistent deformation behavior, and preservation of periodicity: (**a**) after 2% volume compression; (**b**) after 2% volume increase.

**Figure 6:**
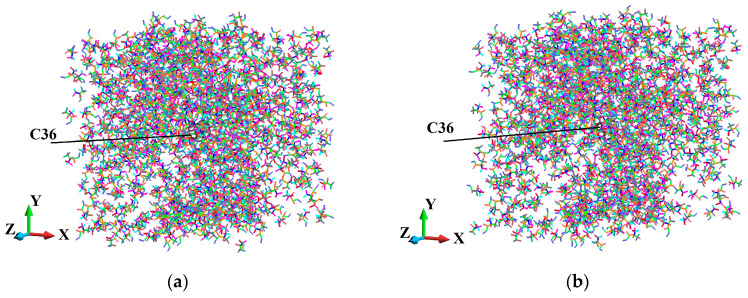
Fully relaxed RVE of C_36_/PMMA representing structural stability, consistent deformation behavior, and preservation of periodicity: (**a**) after 2% volume compression; (**b**) after 2% volume increase.

**Figure 7:**
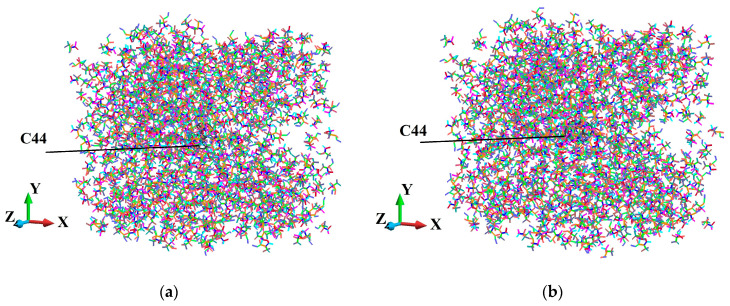
Fully relaxed RVE of C_44_/PMMA representing structural stability, consistent deformation behavior, and preservation of periodicity: (**a**) after 2% volume compression; (**b**) after 2% volume increase.

**Figure 8:**
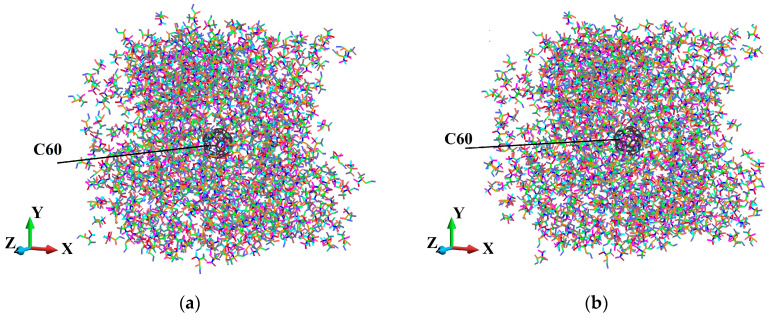
Fully relaxed RVE of C_60_/PMMA representing structural stability, consistent deformation behavior, and preservation of periodicity: (**a**) after 2% volume compression; (**b**) after 2% volume increase.

**Figure 9:**
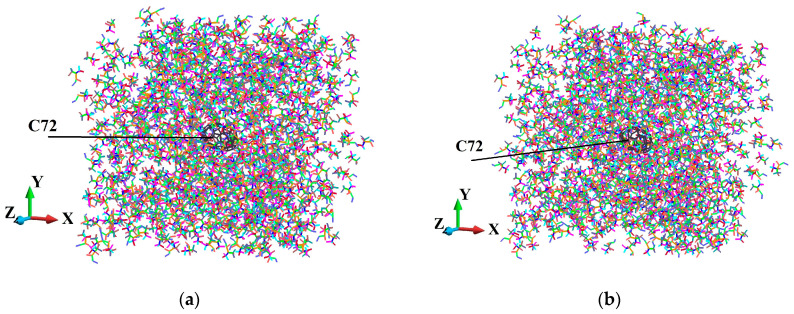
Fully relaxed RVE of C_72_/PMMA representing structural stability, consistent deformation behavior, and preservation of periodicity: (**a**) after 2% volume compression; (**b**) after 2% volume increase.

**Figure 10:**
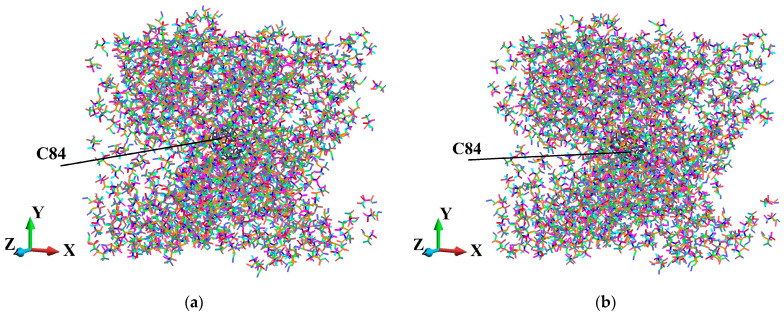
Fully relaxed RVE of C_84_/PMMA representing structural stability, consistent deformation behavior, and preservation of periodicity: (**a**) after 2% volume compression; (**b**) after 2% volume increase.

Figure 5, Figure 6, Figure 7, Figure 8, Figure 9 and Figure 10 illustrate the fullerene-PMMA RVEs after applying volumetric strains for the C_24_, C_36_, C_44_, C_60_, C_72,_ and C_84_ fullerenes, respectively. The view of the systems is towards the *z*-direction. Figure 5, Figure 6, Figure 7, Figure 8, Figure 9 and Figure 10 present the configuration under the compression and tension of the system.

It was observed that the interfacial polymer packing in nanocomposites is strongly influenced by the nanoparticle curvature and confinement effects. In this regard, PMMA chains adjacent to the fullerene surface experience geometric restriction and intermolecular interactions that limit their configurational freedom relative to the bulk-like regions. As a result, the interfacial polymer layer exhibits constrained segmental mobility and enhanced structural organization compared to more distant matrix regions.

Accordingly, it can be seen that upon applying small volumetric strains (±2%), the redistribution of free volume within the amorphous polymer matrix is spatially heterogeneous. Consequently, some low-density pockets observed in visualization snapshots are preferentially located away from the fullerene interface and closer to the periodic cell boundaries. This behavior reflects the higher mechanical constraint and reduced mobility of interfacial polymer segments, which promote more efficient local densification under hydrostatic deformation. In contrast, polymer regions farther from the inclusion retain greater configurational freedom and are, therefore, more susceptible to transient free-volume fluctuations.

It is important to emphasize that the small low-density regions observed in certain visualization snapshots do not correspond to macroscopic cavitation or mechanical instability. The RVE was subjected to both hydrostatic compression (−2%) and hydrostatic expansion (+2%) within the linear elastic regime. Under compression, the global simulation volume decreases, and the overall density increases monotonically, whereas under expansion, the volume increases, and the average density decreases accordingly. In both cases, the system remains mechanically stable and exhibits a reversible elastic response consistent with a positive bulk modulus.

The apparent nanovoid-like features arise from nanoscale free volume redistribution intrinsic to amorphous polymer systems. During the hydrostatic deformation, whether compressive or tensile, PMMA chains undergo heterogeneous segmental rearrangements. Regions with greater configurational freedom, particularly those farther from the fullerene interface, accommodate a larger fraction of volumetric strain and may temporarily exhibit a lower local density. Conversely, interfacial polymer segments, being more constrained, display comparatively reduced density fluctuations.

It should, therefore, be noted that local density fields in atomistic simulations are highly sensitive to spatial coarse-graining and visualization thresholds. The observed rarefaction pockets reflect nonuniform free volume evolution rather than cavitation or structural failure. Such heterogeneous density redistribution under slight hydrostatic strain is a well-established characteristic of amorphous polymers and is fully consistent with the elastic nanoscale deformation of polymer nanocomposites.

Further, the stress distribution in the bonds is presented. The stress is discussed at the end of the process. Table 4 reports the maximum and minimum bond-level stresses allocated to the PMMA matrix of the considered fullerene-PMMA systems.

Table 5 summarizes the maximum and minimum bond-level stresses within the entire system and separately within the fullerene cages under ±2% volumetric strain. In most cases, the largest tensile and compressive stresses occur inside the fullerene nanoparticles, reflecting the high stiffness of the sp^2^ carbon network and its dominant role in resisting hydrostatic deformation. The polymer matrix exhibits comparatively low peak tensile stresses, consistent with its lower elastic modulus and greater segmental flexibility. This stress partitioning demonstrates size-dependent load transfer between the inclusion and the surrounding matrix, where the relative contribution of the nanoparticle to the overall mechanical response increases with particle size.

Also, for the smallest nanoparticle, C_24_, the peak bond-level stresses are observed in the PMMA matrix rather than within the fullerene cage. This behavior can be attributed to the extremely high surface-to-volume ratio of this smallest fullerene. In this regard, the mechanical response becomes surface-dominated rather than volume-dominated. Consequently, load transfer under hydrostatic deformation is distributed more strongly in the surrounding polymer network, leading to higher local stress concentrations in the matrix. As nanoparticle size increases, the relative contribution of the internal sp^2^ carbon network grows, and the fullerene progressively assumes a larger fraction of the applied load, resulting in peak stresses often occurring within the cage structure.

**Figure 11:**
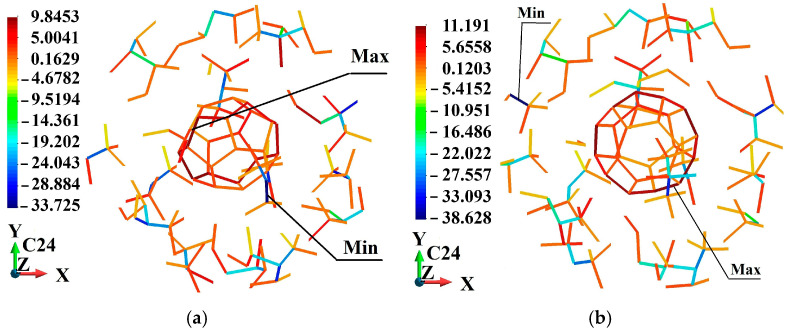
Stress (GPa) distribution in the neighborhood of fullerene C_24_: (**a**) 2% volume decrease; (**b**) 2% volume increase.

**Figure 12:**
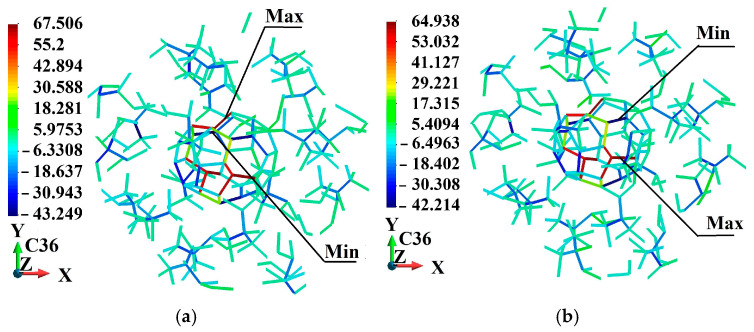
Stress (GPa) distribution in the neighborhood of fullerene C_36_: (**a**) 2% volume decrease; (**b**) 2% volume increase.

**Figure 13:**
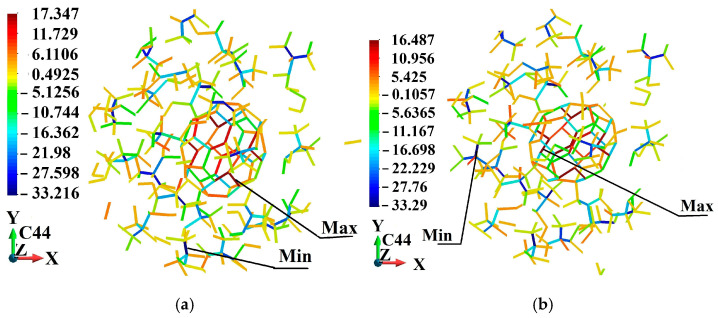
Stress (GPa) distribution in the neighborhood of fullerene C_44_: (**a**) 2% volume decrease; (**b**) 2% volume increase.

**Figure 14:**
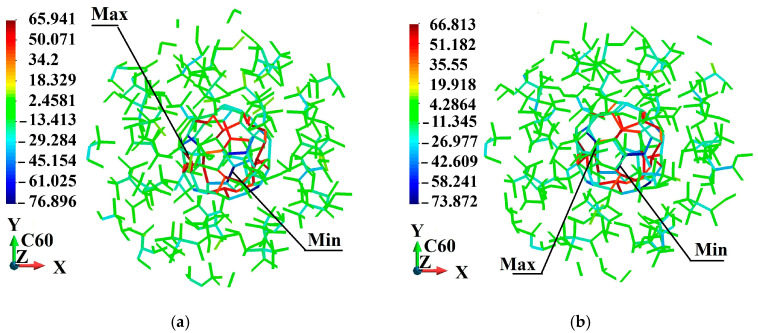
Stress (GPa) distribution in the neighborhood of fullerene C_60_: (**a**) 2% volume decrease; (**b**) 2% volume increase.

**Figure 15:**
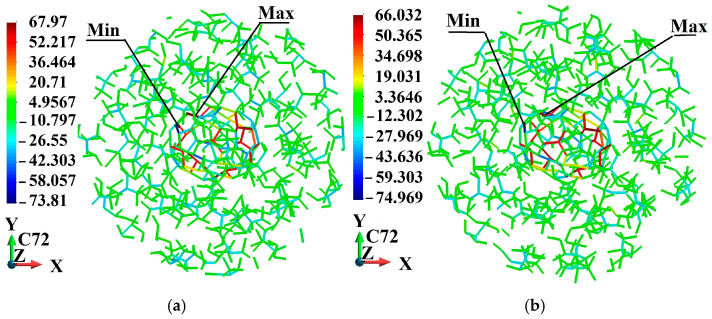
Stress (GPa) distribution in the neighborhood of fullerene C_72_: (**a**) 2% volume decrease; (**b**) 2% volume increase.

**Figure 16:**
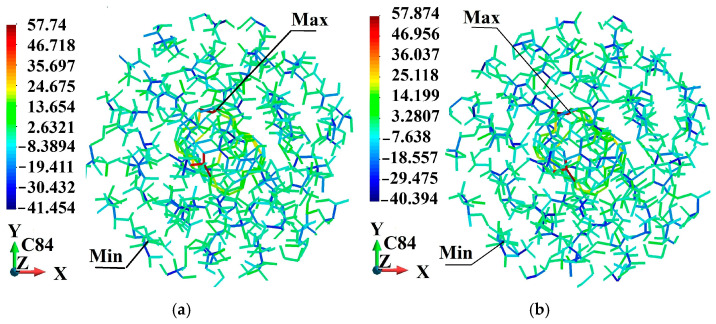
Stress (GPa) distribution in the neighborhood of fullerene C_84_: (**a**) 2% volume decrease; (**b**) 2% volume increase.

The bond-level stress distributions within the fullerene nanoparticles and their immediate surroundings are illustrated in Figure 11, Figure 12, Figure 13, Figure 14, Figure 15 and Figure 16. For clarity and spatial resolution, only a localized region encompassing the fullerene and a surrounding zone extending up to three fullerene radii is visualized. These figures, therefore, highlight the interfacial stress field and near-particle stress gradients rather than the entire RVE. The quantitative global extrema of bond-level stresses across the complete system are already reported separately in Table 5. As observed in the localized visualizations, stresses within fullerene bonds are generally higher than those in the adjacent PMMA matrix, consistent with the higher stiffness of the sp^2^ carbon network and the size-dependent load transfer behavior discussed previously.

### 2.4. Relevance of Bond-Level Stress Distributions to Continuum Modeling

The bond-level virial stress maps reveal that elevated stresses are strongly localized within a narrow interfacial region surrounding the fullerene surface, while stresses in the polymer matrix rapidly decay toward bulk-like values. It is important to emphasize that these values correspond to atomistic virial stress contributions associated with bonded interactions and, therefore, represent localized internal force densities rather than continuum Cauchy stresses. For reference, typical C-C covalent bond rupture forces are on the order of several nanonewtons, corresponding to atomic-scale stresses of approximately 100–300 GPa when normalized by an effective bond cross-sectional area.

The stress of such a range is evaluated in [40,41]. They show the nanotube rupture and the compression of fullerene C_60_, respectively. The stress magnitudes reported in this work under ±2% volumetric strain are substantially below this rupture-equivalent scale, confirming that the observed stress concentrations remain within the elastic regime of the employed force field.

The spatial confinement of elevated bond stresses to a narrow interfacial region provides direct atomistic support for the surface-enhanced continuum formulation adopted in this study, wherein interfacial effects are represented by an effective surface layer possessing distinct elastic properties, consistent with the Gurtin-Murdoch continuum elasticity [17,18]. The clear separation between interfacial and bulk stress regimes supports the assumption of scale separation underlying the micromechanical calibration procedure.

Beyond the elastic parameter extraction, localized stress amplification at the interface may influence the initiation of interfacial damage, bond rearrangement, or nonlinear mechanical response under larger deformations. Although the present study is restricted to the linear elastic regime, the observed interfacial stress heterogeneity highlights the importance of accurately capturing nanoscale interfacial mechanics when predicting long-term durability of polymer nanocomposites.

## 3. Discussion

### 3.1. Interpretation of Bulk Modulus Trends of Fullerene Nanoparticles

The MD-derived bulk modulus values of individual fullerene nanoparticles exhibit a distinct dependence on molecular size and associated geometry. Smaller fullerenes such as C_24_ and C_36_ exhibit lower resistance to volumetric deformation, whereas an appreciable increase in stiffness is observed up to C_60_. Beyond this size, a slight reduction in bulk modulus occurs for C_72_ and C_84_. This non-monotonic trend may be attributed to a combination of atomistic packing density, curvature effects, and cage stability. Fullerenes with smaller diameters possess higher local curvature, which induces bond strain and reduces their elastic stiffness, whereas medium-sized structures attain a more energetically favorable geometry with enhanced rigidity. On the other hand, for larger fullerenes, the increasing delocalization of deformation and structural flexibility results in a marginal decrease in hydrostatic stiffness.

Notably, the adopted Birch-Murnaghan EOS-fitting approach provides a robust means to extract the bulk modulus from atomistic energetics, ensuring that the calculated values represent intrinsic material characteristics rather than numerical artifacts. The strong agreement between the fitted curves and discrete energy-volume data confirms the reliability of the developed MD simulations in capturing volumetric deformation behavior of fullerene molecules.

Although the elastic properties of isolated fullerenes have been reported in the literature previously, the present study computes these quantities within the same atomistic framework used for the nanocomposite simulations to ensure numerical consistency. In other words, these values serve as required inputs for calibrating the surface-enhanced micromechanics model rather than constituting a primary novelty contribution. So, the unified extraction of nanoparticle and composite properties within a single numerical workflow minimizes potential inconsistencies arising from heterogeneous data sources and enables robust multiscale calibration.

### 3.2. Role of Interface Stress and Size Effects in Nanocomposite Stiffening

When fullerenes are embedded within the PMMA matrix, the effective stiffness of the resulting nanocomposite is governed by a coupled contribution from the polymer phase; the fullerene inclusions; and, most critically, the interfacial layer surrounding the nanoparticles. The calibrated micromechanical model clearly highlights that interfacial stresses introduce a significant reinforcing mechanism, modifying the effective bulk modulus beyond classical predictions that neglect nanoscale interface mechanics.

A salient finding is that the surface Lamé modulus remains nearly constant (~18.96–18.98 N/m) across all investigated fullerene sizes. This constancy indicates that the intrinsic mechanical response of the polymer-fullerene interface is size-independent, reflecting a stable interfacial bonding environment. The size dependency of reinforcement, therefore, originates predominantly from geometric factors, specifically the surface to volume ratio of the nanoparticles. Smaller fullerenes have larger interfacial areas for a given volume fraction, leading to stronger surface stress contributions and, consequently, greater modulus enhancement. Conversely, as the particle diameter increases, the interface contribution diminishes, and the composite behavior becomes increasingly governed by bulk material phases.

This observation underscores an essential design implication: nanoscale stiffening in polymer nanocomposites cannot be solely explained by filler stiffness; the interface must be explicitly incorporated for predictive accuracy. The decreasing magnitude of the calibrated dimensionless size parameter with increasing fullerene diameter confirms that interfacial energy effects progressively weaken in larger particles, ultimately approaching classical micromechanics behavior.

### 3.3. Validation and Capability of the Calibrated Micromechanics Model

The agreement between MD results and the predictions of the size-dependent micromechanical framework validates the modeling approach adopted in this study. By embedding MD-calibrated interface parameters into continuum-level constitutive relations, the proposed framework achieves a seamless multiscale correlation between atomistic interaction phenomena and macroscopic nanocomposite properties.

Classical micromechanics theories typically assume perfect bonding and neglect interfacial stresses, leading to a significant underestimation of stiffness in nanostructured systems. The present results demonstrate that introducing surface elasticity parameters yields a more realistic representation of load transfer across the nanoparticle–polymer interface, particularly in systems reinforced with small-diameter inclusions. This confirms that integrating atomistic information into analytical models is essential to ensure physical fidelity in predicting nanoscale composite mechanics.

**Figure 17:**
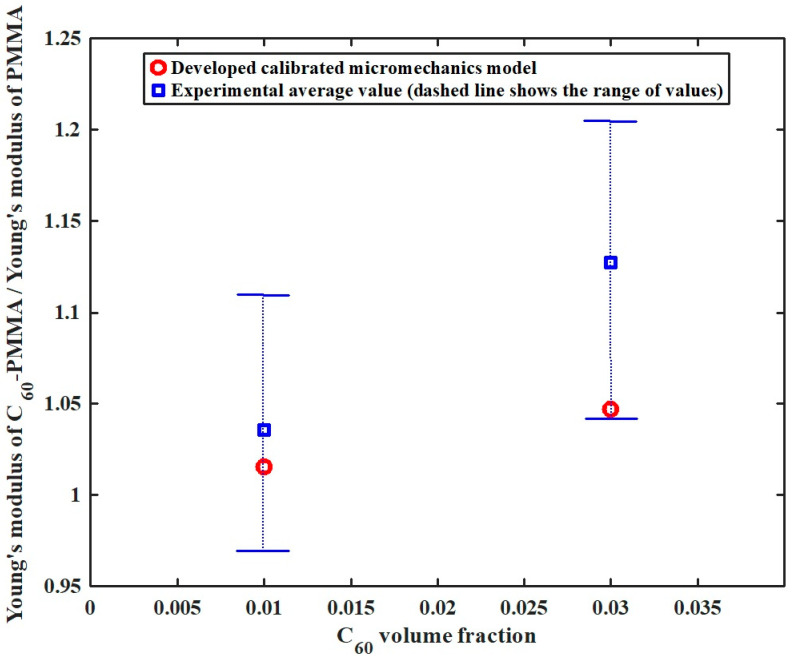
Comparison study of the Young’s modulus of C_60_/PMMA to the Young’s modulus of the PMMA ratio corresponding to different C_60_ volume fractions.

In addition, in order to assess the predictive accuracy of the developed MD-based calibrated micromechanical model, the predicted Young’s modulus of C60/PMMA nanocomposites at various fullerene volume fractions is compared with experimental data reported by Kropka et al. [42]. As shown in Figure 17, the model predictions exhibit good agreement with the experimental measurements, and the resulting Young’s modulus ratios fall within the experimentally reported range. This agreement validates both the accuracy and the predictive capability of the proposed MD-calibrated micromechanical framework for fullerene-PMMA nanocomposites.

### 3.4. Insights from Stress Distribution and Nanoparticle Motion

The stress distribution analysis provides further insight into local deformation mechanisms. Stress fields concentrate primarily within the fullerene cages and their immediate surroundings, whereas the surrounding PMMA experiences comparatively lower stress magnitudes. For some nanoparticle sizes (notably C_36_, C_60_, and C_72_), considerably higher stress magnitudes are observed within fullerene bonds, indicating more effective load transfer from the matrix into the reinforcement phase.

The observed drift of fullerene particles within the polymer during volumetric deformation suggests that the system responds through a combination of particle translation and local matrix rearrangement. Despite different loading conditions, the particles consistently migrate to comparable equilibrium positions, implying stable mechanical coupling between the inclusion and the matrix. Moreover, the tensile stresses in the system are found to be substantially higher in magnitude than compressive stresses, indicating asymmetric deformation resistance and highlighting the nonlinear character of nanoscale hydrostatic loading.

### 3.5. Comparison of Extracted Surface Lamé Modulus with the Literature

While exact surface Lamé modulus values (in N/m) for polymer-carbon interfaces are rare in the literature, the current extracted value of approximately 18.9–19.0 N/m lies within the range of interface stiffness magnitudes reported for carbon-based interfaces and nanoconfined polymer layers [43], and it is consistent with previous atomistic studies of imperfect interfaces in graphene-polymer composites, which report interphase stiffness significantly higher than the bulk polymer but lower than pristine graphene, supporting the physical plausibility of our calibrated surface elastic constants [44]. The present results, therefore, fall within a physically reasonable range and support the validity of the MD-calibrated interface parameters.

The observed size invariance of the surface Lamé modulus further indicates that the intrinsic mechanical response of the polymer-fullerene interface is primarily governed by local chemical interactions rather than nanoparticle curvature. In contrast, the magnitude of its contribution to effective stiffness remains strongly size-dependent through geometric surface-to-volume scaling.

### 3.6. Origin of Heterogeneous Polymer Packing and Nanovoid-like Features After Loading

The heterogeneous density distribution observed after volumetric loading originates from curvature-induced interfacial confinement, combined with strain-dependent free-volume redistribution in the amorphous polymer matrix. The fullerene surface curvature imposes geometric constraints on adjacent PMMA chains, perturbing local segmental conformations and modifying packing relative to the bulk matrix. Such curvature-driven deviations in the interfacial chain organization have been widely reported previously in polymer-nanoparticle systems [45,46,47].

As particle size decreases, the specific interfacial area increases, and a larger fraction of polymer resides within a structurally perturbed interfacial zone. However, interfacial confinement does not necessarily imply reduced density at the nanoparticle surface. In entangled amorphous polymers such as PMMA, geometric restriction and intermolecular interactions near the inclusion can suppress large-scale segmental fluctuations and promote mechanically stabilized packing under deformation.

In addition, the polymer chain mobility and entanglement constraints further influence this behavior. PMMA chains are subject to topological constraints that limit configurational freedom. Near the fullerene surface, confinement and interfacial interactions reduce the amplitude of segmental rearrangements. Consequently, during the application of hydrostatic volumetric strains (±2%), interfacial polymer segments tend to undergo smaller density fluctuations than regions located farther from the inclusion.

The nanovoid-like features observed in visualization snapshots after loading are, therefore, preferentially located in the matrix regions more distant from the fullerene interface, often nearer to the periodic cell boundaries. These regions possess greater configurational freedom and can accommodate volumetric strain through heterogeneous free-volume evolution. Amorphous polymers such as PMMA inherently contain nanoscale free volume arising from chain connectivity and steric constraints. Therefore, under deformation, this free volume redistributes nonuniformly. Regions with higher mobility are more susceptible to transient density depletion, whereas interfacial zones remain mechanically constrained.

Importantly, these local small low-density pockets do not represent macroscopic cavitation or structural failure. The global response remains mechanically stable, with monotonic volume change consistent with the applied hydrostatic strain and a positive bulk modulus. The observed features instead reflect intrinsic nanoscale density fluctuations amplified by the deformation in a heterogeneous polymer–nanoparticle system. The physical consistency of these trends, together with the reversible elastic response and absence of structural instability, further confirms the robustness and accuracy of the developed molecular simulation framework.

### 3.7. Interpretation of Bond-Level Stress Magnitudes

The carried-out stress analysis corresponds to the virial bond-level atomic stresses rather than continuum Cauchy stresses. At the atomistic scale, stress is derived from interatomic force-distance relationships and reflects localized force transmission along individual covalent bonds. Consequently, local bond-level virial stresses can reach tens of gigapascals without implying macroscopic material failure.

Upon application of ±2% volumetric strains and subsequent equilibration, stress redistribution occurs throughout the PMMA matrix and at the interface. Energy minimization and NPT relaxation enable local rearrangements of chain segments, leading to partial homogenization of the force network and reduction of peak local values. This redistribution does not indicate loss of equilibrium. Rather, it reflects the ability of the modeled system to reach a new mechanically stable configuration consistent with the imposed deformation.

Accordingly, in the PMMA matrix, comparatively high compressive bond-level stresses are observed under hydrostatic deformation. These values originate primarily from short-range repulsive interactions in the Lennard-Jones potential and from backbone bond and angle resistance to the associated volumetric contraction. Because virial stresses are normalized over atomic-scale volumes, localized compressive stresses can reach several tens of gigapascals without implying macroscopic yielding. These stresses are spatially heterogeneous and confined to nanoscale regions, reflecting local intermolecular repulsion rather than continuum hydrostatic pressure. The absence of bond rupture or irreversible structural rearrangement confirms that the polymer matrix remains within the elastic regime throughout the applied ±2% volumetric strain range.

Within the fullerene nanoparticles, maximum bond-level stresses within a range of approximately 60–75 GPa can be observed under volumetric loading. These values are consistent with the high stiffness of sp^2^-hybridized carbon networks forming the fullerene cages. Moreover, minor deviations from perfect spherical geometry are visible in atomistic visualizations following loading. This small elastic distortion is physically expected. Although fullerenes exhibit high in-plane stiffness, they are not rigid bodies but finite elastic shells governed by bonded harmonic and dihedral potentials. Under hydrostatic deformation of the simulation cell, the cage undergoes reversible bond stretching and slight angular distortion while preserving structural integrity. The absence of bond rupture, topological defects, or irreversible deformation confirms that the fullerene response remains within the elastic regime.

Overall, the reported bond-level stress analysis should, therefore, be interpreted as localized measures of interatomic force transmission that support the continuum-scale elastic parameter extraction, rather than as direct indicators of macroscopic stress magnitudes. This provides a physically meaningful description of nanoscale load transfer mechanisms within the presented polymer–nanoparticle system. Because the virial formulation is directly derived from the interatomic force field governing the allocated molecular interactions, the computed stresses are fully consistent with the underlying potential energy landscape and the associated mechanical equilibrium conditions. The analysis captures spatial stress heterogeneity at the atomic scale, enabling identification of interfacial stress concentration, matrix response, and nanoparticle deformation within a unified framework. Moreover, the reversible stress evolution under ±2% volumetric strain, the absence of bond rupture or structural instability, and the monotonic global pressure–volume response collectively confirm the internal consistency and numerical stability of the employed MD simulations. Therefore, the presented bond-level stress evaluation constitutes a robust and accurate basis for the mechanistic interpretation and for subsequent continuum-scale elastic parameter calibration.

### 3.8. Implications for MEMS/NEMS Applications

The outcomes of this study provide valuable guidelines for the design of polymer-based nanocomposites for MEMS/NEMS applications. Since device miniaturization inherently amplifies surface-dominated phenomena, the interfacial reinforcing mechanism demonstrated here becomes highly advantageous. Selecting appropriately sized nanoparticles enables the tailoring of elastic properties by exploiting interface-driven stiffening, where smaller fullerenes yield superior reinforcement efficiency at low volume fractions.

However, an optimal balance must be achieved between reinforcement efficiency, processability, and structural stability. While very small nanoparticles enhance interface effects, excessively high surface curvature may introduce local stress concentrations or instability. Therefore, rational composite design requires coordinated control over nanoparticle size, dispersion, interfacial bonding quality, and volume fraction.

Furthermore, the MD-based calibrated surface-enhanced continuum framework presented here is broadly applicable to other amorphous polymer matrices and nanoparticle types, provided that suitable force-field parameters are available to capture the associated bonded and non-bonded interactions. This approach relies on quantifying atomistic stress distributions at the polymer–nanoparticle interface to extract effective interfacial elastic properties, which can then be incorporated into continuum models. Consequently, this methodology can be extended to design nanocomposites with different polymer chemistries or nanoparticle materials, allowing for a predictive evaluation of interface-driven mechanical behavior across a wide range of nanocomposites.

## 4. Materials and Methods

### 4.1. Construction of Employed RVE

To investigate the elastic response of the modeled nanocomposites, we constructed a representative volume element (RVE) that includes a single fullerene embedded in a PMMA matrix. The RVE associated with each fullerene is designed with periodic boundary conditions in all three spatial directions to approximate the bulk behavior, incorporating interface effects. In Figure 4, the employed atomistic RVEs corresponding to each C_24_, C_36_, C_44_, C_60_, C_72_ and C_84_ fullerene nanoparticle surrounded by PMMA chains are displayed.

#### 4.1.1. Atomistic Generation of Fullerene-PMMA RVEs

The initial atomistic RVEs are generated using the PACKMOL package [48] to ensure physically realistic molecular packing while preventing atomic overlap. A cubic simulation box with dimensions of 50 × 50 × 50 Å^3^ is employed. A single fullerene nanoparticle is fixed at the center of the simulation cell at coordinates (25.0 Å, 25.0 Å, 25.0 Å). Surrounding the nanoparticle, an appropriate number of monomer units are randomly packed within the simulation box using a required packing tolerance, resulting in an initial polymer density suitable for subsequent equilibration. Afterwards, to construct realistic polymer chains, a MATLAB R2023a code is employed to process the monomer positions and connectivity, which is then imported into VMD (Visual Molecular Dynamics). Within VMD, the associated bonds, angles, dihedrals, and improper are assigned to generate fully connected PMMA chains, producing a LAMMPS-ready topology with complete bonded interactions for subsequent simulations.

To avoid unphysical overlap between polymer atoms and the fullerene surface during initialization, a spherical exclusion region with an appropriate radius centered at the fullerene is enforced, such that all polymer atoms are constrained to remain outside this region. This procedure ensures controlled interfacial spacing and avoids artificial steric clashes at the initial stage.

This controlled molecular packing strategy ensures the reproducibility of the initial configuration and a consistent interfacial geometry across all simulated RVEs.

#### 4.1.2. Force-Field Description

Bonded interactions within PMMA are modeled using harmonic bond and angle potentials, OPLS dihedral potentials, and CVFF improper potentials. Non-bonded interactions between all atom types (C, H, and O) are described using a Lennard-Jones potential with an appropriate cutoff distance and arithmetic mixing rules. Fullerene–polymer interactions are therefore naturally captured through the same Lennard-Jones formulation without introducing artificial interface constraints. This approach allows interfacial energetics to emerge directly from the molecular force field and ensures consistency across all loading states.

Also, regarding the associated limitations, it is noted that the bonded interactions employ harmonic functional forms, which are most accurate for small deviations around the equilibrium geometries. While this is appropriate for the present small-strain (maximum, 2%) elastic analysis, inharmonic effects at large deformations are not captured. Additionally, the PMMA model represents a linear, non-crosslinked polymer network. Therefore, chemical crosslinking effects and reactive bond rearrangements are not included. The use of fixed OPLS parameters also implies that polarization effects and environment-dependent charge redistribution are neglected. Consequently, while the force field is suitable for evaluating equilibrium structure and small-strain elastic properties, predictions at large strain or under chemical degradation conditions fall outside the intended applicability of the proposed modeling framework.

#### 4.1.3. Equilibrium Protocol

A multi-stage relaxation procedure is applied to each generated RVE prior to mechanical loading in order to remove unfavorable contacts, relax polymer packing, and establish mechanical equilibrium.

At the first stage, the initial configuration is minimized using a conjugate-gradient algorithm until force and energy convergence criteria of 1 × 10^−6^ and 1 × 10^−8^ are satisfied, respectively. This step eliminates residual atomic overlaps and high-energy contacts introduced during packing.

At the second stage, the minimized structure is equilibrated under isothermal and isobaric (NPT) conditions at 300 K and zero external pressure using a Nosé-Hoover thermostat and barostat. The temperature and pressure damping parameters are set to 100 fs and 1000 fs, respectively. An appropriate timestep is used, and the system is equilibrated for the required time, allowing both atomic positions and the simulation box dimensions to relax toward a stable density and zero external pressure. Periodic boundary conditions are applied in all three spatial directions. Thermodynamic quantities, including temperature, pressure, potential energy, and volume, are continuously monitored to confirm convergence.

Finally, a second energy minimization is also performed to eliminate residual thermal fluctuations and ensure a mechanically stable reference configuration suitable for static energy-volume analysis.

#### 4.1.4. Incorporation of the Interface Effect

The inclusion of the fullerene nanoparticle alters the local polymer density and interaction energies. To capture these effects, the RVE size in each case is selected so that the polymer region around the fullerene extends far enough to reproduce bulk-like packing beyond the interfacial zone. In this way, the total potential energy of the system during volumetric deformation is composed of contributions from

- Intramolecular deformation of PMMA chains;
- Intermolecular interactions within the polymer matrix;
- The elastic distortion of the fullerene itself;
- Interfacial interaction energy between the fullerene nanoparticle and PMMA.

Since the same interaction parameters are consistently used across all strain states, the fitted bulk modulus effectively reflects the combined effects of the polymer, the fullerene, and their interface. This approach provides a direct measure of the nanocomposite’s effective bulk modulus at the molecular scale.

#### 4.1.5. Applying Volumetric Strain

To probe the elastic response under hydrostatic loading, the simulation cell is isotropically strained through the uniform scaling of all three box lengths. In practice, this is achieved in LAMMPS by remapping the simulation box dimensions while scaling the atomic coordinates proportionally. The applied strain states spanned both compression and expansion relative to the equilibrium cell, typically within ±2% of the equilibrium volume.

Each strained configuration is subsequently relaxed using energy minimization until the residual forces and stresses converge below a stringent threshold. Accordingly, a short low-temperature NVT relaxation is performed to relieve any local atomic overlaps introduced by coordinate remapping. Subsequently, a robust FIRE energy minimization is applied until strict force and energy convergence criteria are satisfied. Only the fully relaxed equilibrium volume and potential energy are recorded for the associated equation-of-state fitting. This procedure ensures that the recorded energies represent the true minimum-energy states at each prescribed volume. The potential energy and final box dimensions are written to the output for further analysis. Because periodic boundary conditions are maintained, the remapped box represents a homogeneous hydrostatic strain condition across the RVE.

#### 4.1.6. Volume Fractions of Constituent Phases

A fundamental parameter for characterizing nanocomposites is the volume fraction of each constituent phase, as it directly affects the material’s mechanical, thermal, and transport properties. For systems containing nanoparticles dispersed within a polymer matrix, the volume fraction quantifies the relative volume occupied by the filler and matrix, which, in turn, strongly influences the macroscopic effective behavior. In experimental studies, volume fractions are usually controlled by adjusting the mass ratio of components during synthesis or processing. In atomistic simulations, the number of molecules of each species is specified explicitly. However, converting this molecular information into an accurate volume fraction requires a careful approach, particularly for nanoparticles that are discrete and occupy space in a non-continuous manner.

In the present study, the volume fraction of fullerene nanoparticles is calculated by explicitly considering the actual occupied volume of the nanoparticles within the RVE, rather than relying solely on bulk density or idealized geometric assumptions. This approach will be more useful for carrying out the comparison study associated with the developed micromechanics model in the next section. Each fullerene molecule is treated as occupying a finite, well-defined volume based on its van der Waals radius or molecular packing. The total occupied volume of all nanoparticles is then considered:

(3) $$\vartheta_{f}=V_{fullerence},$$

where $V_{fullerence}$ is the effective volume occupied by a single fullerene molecule. Similarly, the polymer matrix occupies the remaining volume within the RVE, denoted as $\vartheta_{m}$. If the RVE volume $V_{RVE}$ is known, the polymer volume can be expressed as

(4) $$\vartheta_{m}=V_{RVE}-\vartheta_{f}\;,$$

As a consequence, the volume fraction of the fullerene phase is then expressed as

(5) $$V_{f}=\frac{\vartheta_{f}}{\vartheta_{f}+\vartheta_{m}}\;\;.$$

This method ensures that the volume fraction reflects the actual physical space occupied by the nanoparticles, accounting for their discrete molecular sizes and avoiding assumptions about idealized shapes or bulk packing. The corresponding weight fraction can still be obtained for comparison with experimental formulations using

(6) $$W_{f}=\frac{M_{f}}{M_{f}+M_{m}}\;,$$

where $M_{f}$ is the molar mass of a fullerene molecule, and $M_{m}$ is the total mass of the polymer in the RVE.

An important advantage of this occupied-volume approach is its accuracy and consistency across different RVEs. Because it uses molecular-scale information directly, it can be applied regardless of the number of polymer chains, chain lengths, or nanoparticle loadings, providing a reliable foundation for studying, in a physically meaningful way, how nanoparticle concentration affects mechanical properties, such as bulk modulus and stiffness.

It should be noted that the atomistic simulations are intentionally restricted to low fullerene volume fractions (up to 3%) to isolate interfacial stress effects and avoid complications associated with particle-particle interactions, clustering, and finite-size artifacts inherent to small periodic simulation cells. While direct MD simulation of higher volume fractions would be computationally prohibitive, the calibrated surface-enhanced micromechanics model enables efficient prediction of the effective bulk modulus for arbitrary fullerene sizes and volume fractions once the interfacial parameters are identified. Consequently, the developed numerical framework provides predictive capability beyond the explicitly simulated volume fraction range.

#### 4.1.7. Bulk Modulus Extraction

The series of minimized energies, E(V), is plotted as a function of the simulation cell volume. To extract the bulk modulus, these data are fitted to the third-order Birch-Murnaghan equation of state, as described mathematically in the previous section. Fitting is performed using nonlinear least-squares optimization.

### 4.2. Description of the Micromechanics Model

**Figure 18:**
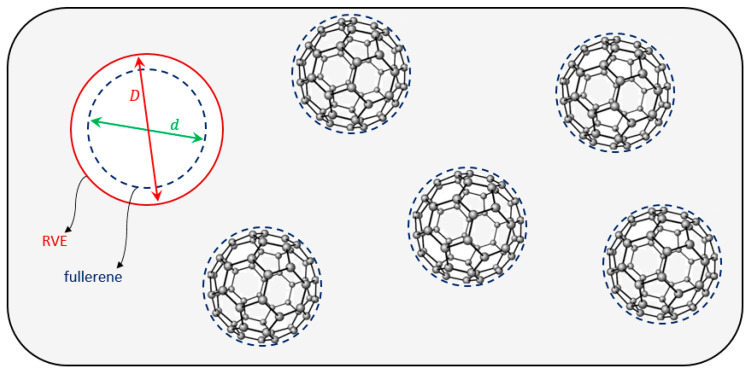
Schematic representation of multi-phase RVE for the nanocomposite randomly reinforced by fullerene nanoparticles.

For the considered nanocomposite material randomly reinforced by fullerene nanoparticles, a spherical multi-phase RVE is taken into account, consisting of two phases: an interior phase (fullerene nanoparticle including surface stress effect) and matrix phase, as shown in Figure 18.

Consequently, the effective material properties of randomly reinforced nanocomposites are estimated, incorporating the surface stress effect associated with the fullerene nanoparticles. In the selected spherical RVE, the parameter d represents the diameter of the fullerene nanoparticle, and D denotes the total diameter. In other words, the fullerene volume fraction, $V_{f}$, can be obtained as follows:

(7) $$V_{f}={\left(\frac{d}{D}\right)}^{3}\;.$$

On the basis of the strain energy-based homogenization approach, the volume-averaged strain energy associated with an elastic medium, ϑ, can be expressed as

(8) $$U_{\omega }=\frac{1}{2V_{\omega }}\int_{V_{\omega }}\left\{\sigma_{ij}\varepsilon_{ij}\right\}dV_{\omega }\;,$$

in which $\sigma_{ij}$ and $\varepsilon_{ij}$ stand for, respectively, the pointwise stress and strain fields associated with the considered elastic medium. By assuming a uniform strain field $\varepsilon_{ij}^{0}$ applied to the elastic medium, one will have

(9) $$U_{\omega }=\frac{\varepsilon_{ij}^{0}}{2V_{\omega }}\int_{V_{\omega }}\sigma_{ij}dV_{\omega }\;.$$

An average stress field is taken into account for the elastic medium as follows:

(10) $${\bar{\sigma }}_{ij}=\frac{1}{V_{\omega }}\int_{V_{\omega }}\sigma_{ij}dV_{\omega }\;.$$

As a consequence, it yields

(11) $$U_{\vartheta }=\frac{1}{2}{\bar{\sigma }}_{ij}\varepsilon_{ij}^{0}\;.$$

By making an identical homogenous counterpart for the selected multi-phase RVE, the stored strain energy takes the following form:

(12) $$U_{\omega }=\frac{1}{2}\sigma_{ij}^{*}\varepsilon_{ij}^{0}=\lambda \varepsilon_{kk}^{0}\delta_{ij}+2\mu \varepsilon_{ij}^{0}\;,$$

where λ and μ denote the effective Lamé constants of the homogeneous counterpart of the RVE. In addition, the stored strain energy in the introduced multi-phase RVE includes two parts, as follows:

(13) $$U_{\vartheta }=U_{m}+U_{f}\;,$$

in which the volume-averaged strain energy associated with the matrix phase is expressed as

(14) $$U_{m}=\frac{1}{2}{\bar{\sigma }}_{ij}^{m}\varepsilon_{ij}^{0}\;,$$

where ${\bar{\sigma }}_{ij}^{m}$ refers to the average stress field within the matrix phase. On the other hand, for the interior phase, one will have

(15) $$U_{f}=\frac{1}{2}{\bar{\sigma }}_{ij}^{f}\varepsilon_{ij}^{0}+\frac{1}{2}\tau_{ij}\varepsilon_{ij}^{0}\;,$$

where $\lambda_{s}$ and $\mu_{s}$ represent the surface Lamé constants.

As a consequence, the average stress field of the identical homogenous counterpart for the selected multi-phase RVE can be rewritten in the following form:

(16) $$\sigma_{ij}^{*}=\left[1-{\left(\frac{d}{D}\right)}^{3}\right]\sigma_{ij}^{m}+{\left(\frac{d}{D}\right)}^{3}\left(\sigma_{ij}^{f}+\tau_{ij}\right)\;,$$

in which

(17) $$\sigma_{ij}^{m}=\lambda_{m}\varepsilon_{kk}^{m}\delta_{ij}+2\mu_{m}\varepsilon_{ij}^{m},\;\sigma_{ij}^{f}=\lambda_{f}\varepsilon_{kk}^{f}\delta_{ij}+2\mu_{f}\varepsilon_{ij}^{f}\;,$$

where $\lambda_{m}$ and $\mu_{m}$ are the Lamé constants for the matrix phase, and $\lambda_{f}$ and $\mu_{f}$ denote the Lamé constants associated with the fullerene nanoparticles.

The strain fields related to the interior phase of the considered multi-phase RVE, including $\varepsilon_{ij}^{f}$ and $\varepsilon_{ij}^{s}$, can be given as follows:

(18) $$\varepsilon_{ij}^{f}=A_{ijkl}\varepsilon_{kl}^{0}\;,\;\;\;\;\;\;\varepsilon_{ij}^{s}=B_{ijkl}\varepsilon_{kl}^{0}\;,$$

in which $A_{ijkl}$ and $B_{ijkl}$ refer to the fourth-order strain concentration tensors.

In this regard, the effective bulk modulus of the multi-phase RVE can be extracted in terms of the elastic properties associated with its constituent phases, as follows:

(19) $$K=K_{m}+{\left(\frac{d}{D}\right)}^{3}\left(K_{f}-K_{m}\right)A_{k}+{\left(\frac{d}{D}\right)}^{3}K_{s}B_{k}\;,$$

where $A_{k}.\;A_{g}$ and $B_{k}.\;B_{g}$ are scalar forms of the $A_{ijkl}$ and $B_{ijkl}$ concentration tensors associated with the considered multi-phase RVE, which can be derived based on the micromechanical model presented by Duan et al. [49] for composites with spherical inhomogeneity. As a result, the effective bulk modulus of the considered multi-phase RVE attributed to the modeled fullerene/PMMA nanocomposites can be achieved in the following forms [49]:

(20) $$K=\frac{3K_{f}\left[4V_{f}\mu_{m}+3K_{m}\right]+2\mu_{m}\left[4V_{f}\mu_{m}K_{s}^{*}+3K_{m}\left(2-2V_{f}+K_{s}^{*}\right)\right]}{3\left[3\left(1-V_{f}\right)K_{f}+3V_{f}K_{m}+2\mu_{m}\left(2+K_{s}^{*}-V_{f}K_{s}^{*}\right)\right]}\;,$$

in which $K_{f}$ and $K_{m}$, in order, represent the bulk modulus of the fullerene and PMMA phases; $\mu_{m}$ denotes the shear modulus of PMMA; and $K_{s}^{*}$ is a dimensionless size-dependent parameter allocated to the interface effect, which can be defined as follows [49]:

(21) $$K_{s}^{*}=\frac{2K_{s}}{\mu_{m}d}$$

where $K_{s}=2\left(\mu_{s}+\lambda_{s}\right)$ stands for the surface Lamé modulus attributed to the interface effect.

By inserting the obtained values K, $K_{f}$, and d via the carried-out MD simulation in Equation (21), and assuming $K_{m}=2.4\;GPa$ and $\mu_{m}=1.185\;GPa$ [50], the calibrated values of the size-dependent dimensionless parameter, $K_{s}^{*}$, as well as the surface Lamé modulus, can be extracted corresponding to each type of the considered fullerene nanoparticle.

## 5. Conclusions

The current study presented a combined MD and micromechanical modeling approach to investigate the influence of interfacial stresses on the bulk modulus of fullerene/PMMA nanocomposites. The key findings can be summarized as follows:

- Size-dependent Interfacial Effects: The dimensionless interface parameter, $K_{s}^{*}$, decreases with increasing fullerene nanoparticle size, reflecting the reduced contribution of surface stresses in larger particles due to lower surface-to-volume ratios. Smaller nanoparticles, therefore, exhibit a more pronounced interfacial effect, significantly enhancing the effective bulk modulus of the nanocomposite.
- Constancy of Surface Lamé Modulus: The surface Lamé modulus, $K_{s}$, remains nearly constant (~18.96–18.98 N/m) across all nanoparticle types, indicating that the intrinsic stiffness of the polymer–fullerene interface is mainly independent of the particle size and governed primarily by the local interfacial bonding characteristics.
- Physically Consistent Bond-Level Stress Analysis: Virial bond-level stresses provide mechanistic insights into nanoscale load transfer and interfacial force transmission, and they remain within the elastic regime of covalent interactions. The absence of bond rupture, irreversible deformation, or instability confirms the mechanical robustness and internal consistency of the employed MD simulations.
- Validation of Micromechanical Model: The calibrated micromechanical model, incorporating the size-dependent interfacial parameter, accurately captures the influence of interfacial stresses on the composite’s bulk modulus, bridging atomistic interfacial phenomena with continuum-scale mechanical predictions.
- Quantitative Interface Parameter Extraction: The proposed MD-based calibrated framework enables direct extraction of physically meaningful surface elastic constants for polymer–fullerene interfaces, providing a transferable parameter set for continuum modeling of nanoscale composites.
- Implications for Material Design: The achieved results underscore the critical importance of nanoparticle size and interfacial effects in designing polymer nanocomposites for MEMS/NEMS applications. By optimizing the particle size and volume fraction, stiffness enhancement derived from interfacial contributions can be maximized.

Therefore, this research work demonstrates that surface stress effects are a dominant factor in nanoscale mechanical reinforcement and provides a physically consistent, multiscale modeling methodology for predicting the effective elastic properties of polymer nanocomposites. Beyond reproducing atomistic trends, the developed MD-based calibrated micromechanics framework enables the predictive evaluation of elastic behavior across nanoparticle sizes and volume fractions without additional atomistic simulations. This multiscale capability provides a practical pathway for linking nanoscale interfacial mechanics to macroscopically measurable elastic properties, supporting the rational design of surface-dominated nanocomposites.

## Figures and Tables

**Table 1:** Obtained bulk modulus of the considered fullerene nanoparticles via MD simulation.

Type of Fullerene Particle	Approximate Radius (nm)	Bulk Modulus (GPa)
C_24_	0.251	137.97
C_36_	0.304	189.42
C_44_	0.322	310.69
C_60_	0.355	401.98
C_72_	0.396	393.11
C_84_	0.435	370.83

**Table 2:** Obtained bulk modulus of the modeled fullerene/PMMA nanocomposites via MD simulation.

Type of Composite	*V_f_* Based on the Considered RVE (%)	Bulk Modulus (kPa)
C_24_/PMMA	0.053	2,402,066.265
C_36_/PMMA	0.094	2,403,680.587
C_44_/PMMA	0.112	2,404,412.019
C_60_/PMMA	0.150	2,405,924.708
C_72_/PMMA	0.208	2,408,218.107
C_84_/PMMA	0.276	2,410,905.711

**Table 3:** Extracted calibrated value of surface bulk modulus allocated to the interface effect in fullerene/PMMA nanocomposites.

Type of Fullerene Particle	*V_f_* Based on the Considered RVE (%)	Calibrated Value of $K_{s}^{*}$	Calibrated Value of $K_{s}$ (N/m)
C_24_	0.053	63.748	18.961
C_36_	0.094	52.665	18.972
C_44_	0.112	49.713	18.969
C_60_	0.150	45.127	18.984
C_72_	0.208	40.440	18.977
C_84_	0.276	36.822	18.981

**Table 4:** Maximum and minimum bond-level stresses in the matrix corresponding to −2.0% and +2.0% applied volumetric strains.

Type of Fullerene	Applied Strain (%)	Maximum Bond Stress (GPa)	Minimum Bond Stress (GPa)
C_24_	−2.0	13.793	−44.334
C_24_	+2.0	13.473	−43.594
C_36_	−2.0	12.348	−45.118
C_36_	+2.0	11.871	−42.023
C_44_	−2.0	11.521	−51.259
C_44_	+2.0	11.071	−50.905
C_60_	−2.0	9.798	−42.526
C_60_	+2.0	13.742	−43.479
C_72_	−2.0	11.698	−47.407
C_72_	+2.0	14.326	−46.329
C_84_	−2.0	11.878	−43.667
C_84_	+2.0	12.843	−46.387

**Table 5:** Maximum and minimum bond-level stresses in the system and in the fullerenes corresponding to −2.0% and +2.0% applied volumetric strains.

Type of Fullerene	Applied Strain (%)	Maximum Bond Stress in the System (GPa)	Minimum Bond Stress in the System (GPa)	Maximum Bond Stress in Fullerene (GPa)	Minimum Bond Stress in Fullerene (GPa)
C_24_	−2.0	13.793	−44.334	9.845	−1.818
C_24_	+2.0	13.473	−43.594	11.191	−2.905
C_36_	−2.0	67.506	−45.118	67.506	−43.249
C_36_	+2.0	64.938	−42.214	64.938	−42.214
C_44_	−2.0	17.347	−51.259	17.347	−19.032
C_44_	+2.0	16.487	−50.905	16.487	−19.822
C_60_	−2.0	65.941	−76.896	65.941	−76.896
C_60_	+2.0	66.813	−73.872	66.813	−73.872
C_72_	−2.0	67.970	−73.810	67.970	−73.810
C_72_	+2.0	66.032	−74.969	66.032	−74.969
C_84_	−2.0	57.740	−43.667	57.740	−29.557
C_84_	+2.0	57.844	−46.387	57.874	−28.493

## Data Availability

The data supporting the findings of this study consist of the output dump files generated from the numerical simulations, which can be made available by the corresponding author upon reasonable request. The main computational codes are not publicly available due to internal research constraints.

## Abbreviations

The following abbreviations are used in this manuscript:

MEMS	Micro-electromechanical systems
NEMS	Nano-electromechanical systems
RVE	Representative volume element
MD	Molecular dynamics
LAMMPS	Large-Scale Atomic/Molecular Massively Parallel Simulator
AIREBO	Adaptive Intermolecular Reactive Empirical Bond-Order potential
EOS	Equation of state
PMMA	Poly(methyl methacrylate)
